# Correlation of platelet–lymphocyte ratio, systemic immune-inflammation index, and mean platelet volume with disease activity in rheumatoid arthritis: A monocentric and retrospective study

**DOI:** 10.1097/MD.0000000000043667

**Published:** 2025-08-08

**Authors:** Haihua Wu, Dan Yin, Chenyang Ma, Xiufang Chen

**Affiliations:** a Department of Rheumatology, Jiangnan Hospital Affiliated to Zhejiang Chinese Medical University, Hangzhou, China; b Department of Internal Medicine, The Second People’s Hospital, Hangzhou, China.

**Keywords:** disease activity, mean platelet volume, platelet-to-lymphocyte ratio, rheumatoid arthritis, systemic immune-inflammation index

## Abstract

This study analyzes the platelet–lymphocyte ratio (PLR), systemic immune-inflammation index (SII), and mean platelet volume (MPV) in the evaluation of disease activity in patients with rheumatoid arthritis (RA). The clinical data of patients with RA and healthy controls, including complete blood count, C-reactive protein (CRP), erythrocyte sedimentation rate (ESR), anti-cyclic citrullinated peptide antibody (anti-CCP antibody), and rheumatoid factor (RF), were retrospectively analyzed. The SII, PLR, and MPV were collected and calculated for the RA. Pearson correlation analysis was used to estimate the correlation between hematological indices and DAS28-ESR. Receiver-operating characteristic (ROC) curve analysis was performed to assess the value of these indices in differentiating active RA from RA remission. The results revealed that SII and PLR were elevated and MPV was markedly decreased in patients with RA compared to healthy controls (all *P* < .05). Statistical significance was observed in SII and MPV, but not in PLR, between the active RA patients and the remission RA group. SII and PLR were positively correlated with DAS28-ESR and DAS28-CRP, whereas MPV was negatively correlated with DAS28-CRP but not with DAS28-ESR. The ROC curve showed that the SII and MPV were helpful in assessing disease activity in RA. SII and MPV were correlated with the DAS28 score and may be used as complementary tools to evaluate disease activity in patients with RA.

## 1. Introduction

Rheumatoid arthritis (RA) is an autoimmune inflammatory disorder characterized by persistent synovitis, progressive joint destruction, and extra-articular manifestations.^[1]^ Affecting approximately 0.5% to 1% of the global population,^[2]^ RA not only causes functional disability and diminished quality of life but also elevates the risk of systemic complications, including cardiovascular events and interstitial lung disease. Progressive RA disability severely impacts patients’ physical function and generates considerable economic strain.^[3]^ Evidence shows that inflammation is the primary determinant and critical pathogenic mechanism in RA, and uncontrolled inflammation drives pathogenesis in connective tissue disorders,^[4]^ often leading to severe disability and elevated mortality. So using reliable biomarkers to measure inflammation in RA is essential for monitoring disease activity. Current clinical assessment of RA disease activity relies on composite indices (e.g., DAS28, CDAI), acute-phase reactants (CRP, ESR), and imaging techniques. However, these methods face limitations such as operational complexity, cost constraints, or delayed reflection of inflammatory dynamics. There is an urgent need to develop new inflammatory biomarkers with enhanced precision, practicality, and cost-efficiency.

In clinical practice, complete blood count analysis enables infection and inflammation assessment. This utility stems from platelets and WBCs within chronically inflamed tissues, which trigger chemokine, protease, angiogenic factor, and cytokine production. As a result, peripheral blood cell parameters enable practical inflammatory activity monitoring. These hematological changes therefore provide a basis for evaluating inflammatory activity. Evidence suggests that platelet–lymphocyte ratio (PLR) and SII, integrating dynamic changes in platelets,^[5]^ neutrophils,^[6]^ and lymphocytes,^[7]^ effectively reflect pro-inflammatory states and immune imbalance in RA. The Systemic Immune-Inflammation Index (SII), as a parameter for assessing systemic inflammatory response, has been reported to be related to the prognosis and progression of psoriatic arthritis. On the other hand, mean platelet volume (MPV) quantifies the average size of circulating platelets,^[8]^ serving as a dynamic biomarker of platelet activity and hematopoietic function. Its clinical utility extends across inflammation, thrombosis, and immune regulation.^[9]^ This study aims to systematically analyze the associations of PLR, SII, and MPV with RA disease activity – encompassing clinical symptoms, serological markers – to establish their clinical value as auxiliary biomarkers and provide evidence-based support for optimizing inflammatory monitoring and stratified management in RA.

## 2. Materials and methods

### 2.1. Study subjects

A total of 216 patients diagnosed with RA were preliminarily selected from Jiangnan Hospital Affiliated to Zhejiang Chinese Medical University, Hangzhou, China, from January 1, 2022, to October 31, 2023. The selection criteria were as follows: age ≥ 18 years; fulfillment of the classification criteria for RA as proposed by the American College of Rheumatology (ACR)/European League Against Rheumatism (EULAR) in 2010.^[10]^ The exclusion criteria were as follows: age younger than 18 years or older than 90 years; concomitant with other autoimmune diseases, such as Sjogren syndrome and systemic lupus erythematosus; malignant diseases; acute infection or chronic inflammation status; and hematological diseases or had received blood transfusion during the past 4 months. The medical records of 216 patients were reviewed by 2 rheumatology specialists. Among them, 35 cases did not meet the 2010 ACR/EULAR classification criteria, 30 cases lacked clinical or laboratory data, 13 cases were complicated by other rheumatic diseases, 3 cases had infections, and 3 were complicated by tumors. In total, 132 patients with RA were enrolled in this study. Ninety age- and sex-matched healthy subjects who underwent routine physical examinations at the same hospital were used as healthy controls (HCs).

### 2.2. Data extraction

Demographic, clinical, and laboratory data were extracted from the electronic medical records, including age, sex, disease duration (years), swollen joint count (SJC), tender joint count (TJC), patient global health assessment (PGA), white blood cell (WBC), neutrophil (N), lymphocyte (L), platelet (P), MPV, erythrocyte sedimentation rate (ESR), C-reactive protein (CRP), anti-cyclic citrullinated peptide antibody (anti-CCP antibody, IU/mL), and rheumatoid factor (RF, IU/mL). PGA was assessed using the 100 mm visual analogue scale (VAS). In routine clinical practice, the most commonly used indices to evaluate disease activity in RA are Disease Activity Score 28 joints (DAS28),^[11]^ which are multidimensional instruments that utilize TJCs and SJC, patient and physician global health assessment of disease activity, and acute-phase reactants. In this study, we used DAS28-ESR to evaluate the disease activity of RA patients and arbitrarily classified them into 2 groups: remission and active. According to the local procedures of routine physical examination, only demographic data and hemograms were available for the analysis of the HCs. This study was approved by the Ethics Committee of Jiangnan Hospital Affiliated to Zhejiang Chinese Medical University and conducted in accordance with the Declaration of Helsinki Informed consent.

### 2.3. Blood cell-based indexes calculation

Total and differential WBCs (neutrophils and lymphocytes) and platelets were assessed in peripheral venous blood obtained from each participant. The following hematological indices were derived from the absolute leukocyte (WBC), neutrophil (N), lymphocyte (L), and platelet (P) counts: the platelet-to-lymphocyte ratio (PLR) = P/R; the systemic immune-inflammation index (SII) = platelet × neutrophil/lymphocyte ratio.

### 2.4. Statistical analysis

IBM SPSS Statistics 27 (SPSS, Chicago) was used for data analysis. The results are presented as mean ± SD or median (interquartile range) for continuous variables and number (% of cases) for categorical variables. The Kolmogorov–Smirnov test was used to determine the normality of the data distribution. Statistical differences between groups were assessed using Student *t* test, Wilcoxon rank-sum test, or Kruskal–Wallis test, as appropriate. The chi-square test or Fisher exact test was performed to compare categorical variables. Pearson correlation analysis was used to estimate the correlation between hematological indices and disease activity variables associated with RA. The ability of the different hematological indices to discriminate between active and remission in RA was assessed using receiver-operating characteristic (ROC) curve analysis and expressed as the area under the curve with 95% confidence intervals (95% CI). The optimal cutoff values of hematological indices for sensitivity and specificity were explored using ROC analysis based on Youden index. *P* ≤ .05.

## 3. Results

### 3.1. Basic characteristics of the study samples

The clinical and laboratory characteristics of patients with RA and HCs are shown in Table 1. The study included 132 patients with RA and 90 healthy subjects. The age and sex distributions were similar between patients with RA and HCs (*P* > .05). As expected, female sex was the main factor according to the RA epidemiology. The mean age of RA patients and HCs was 58.30 ± 13.07 years and 60.82 ± 14.22 years, respectively. The mean disease duration of RA patients was 3 (1,6) years, and the levels of RF and anti-CCP antibody were 86.2 (32.48, 158.45) IU/mL and 182 (24.3, 200) IU/mL, respectively. The mean DAS28-ESR and DAS28-CRP were 3.47 (2.53, 4.7) and 2.94 (2.20, 4.20), respectively. The mean CRP level was 3.51 (1.28, 13.05) mg/L and the mean ESR level was 22 (12, 41) mm/h.

**Table 1:** Comparison between RA and HCs regarding demographic and laboratory data.

Characteristics	RA (132)	HCs (90)	*P*-value
Age (yr)	58.30 ± 13.07	60.82 ± 14.22	.17
Sex (F/M)	101/31	76/14	.15
WBC (10^9^/L)	6.51 (5.17, 7.85)	6.07 (5.24, 7.51)	.384
PLR	145.25 (112.7, 199.59)	80.44 (55.68, 119.27)	<.001
PLT (10^12^/L)	247.5 (208, 299)	198.5 (163.25, 233.75)	<.001
SII	592.76 (396.35, 895.74)	447.2 (317.42, 570.26)	<.001
MPV (fL)	8.9 (8.18, 9.33)	10.1 (9.43, 10.6)	<.001
L (10^9^/L)	1.7 (1.2, 2)	1.6 (1.33, 2)	.87
N (10^9^/L)	4 (3, 5.7)	3.7 (3.03, 5)	.28

HCs = healthy controls, MPV = mean platelet volume, PLR = platelet-to-lymphocyte ratio, PLT = platelet, RA = rheumatoid arthritis, SII = systemic immune-inflammation index, WBC = white blood cell.

### 3.2. Hematological indices of RA patients and HCs

As shown in Table 1, PLR, PLT, and SII were markedly elevated (*P* < .01), and MPV was significantly decreased (*P* < .01) in RA patients compared with HCs. There was no statistical difference between these 2 groups in WBC, lymphocyte count (L), and neutrophil count (N) (*P* all > 0.05) (Table 1).

### 3.3. Correlation between hematological indices and disease activity scores and clinical indicators

In our study, we found that SII and PLR were positively correlated with DAS28-CRP, DAS28-ESR, CRP, ESR, VAS, SJC, and TJC (Table 3). MPV was negatively correlated with DAS28-CRP, CRP, and ESR but had no obvious correlation with DAS28-ESR, VAS, SJC, and TJC. However, all the correlations were relatively weak or mild (*R* < 0.5). All hematological indices showed no correlation with anti-CCP antibodies or RF (Table 2).

**Table 2:** Correlation between hematological indices and disease activity indices and clinical indices in rheumatoid arthritis.

Varies	SII		PLR		MPV		PLT	*P*
	*r*	*P*	*r*	*P*	*r*	*P*	*r*	
Das28CRP	0.32	<.01	0.25	<.01	−0.19	<.05	0.37	<.01
Das28ESR	0.33	<.01	0.30	<.01	−0.16	.08	0.39	<.01
CRP	0.41	<.01	0.42	<.01	−0.26	<.01	0.55	<.01
ESR	0.34	<.01	0.37	<.01	−0.2	<.05	0.39	<.01
VAS	0.31	<.01	0.27	<.01	−0.11	.2	0.34	<.01
SJC	0.26	<.01	0.26	<.01	−0.13	.14	0.35	<.01
TJC	0.24	<.01	0.25	<.01	−0.15	.08	0.34	<.01
Anti-CCP	−0.03	.73	0.04	.63	0.01	.99	0.06	.49
RF	0.03	.74	−0.01	.88	−0.11	.23	0.15	.10

Anti-CCP = anti-cyclic citrullinated peptide antibody, CRP = C-reactive protein, ESR = erythrocyte sedimentation rate, MPV = mean platelet volume, PLR = platelet-to-lymphocyte ratio, PLT = platelet, RF = rheumatoid factor, SII = systemic immune-inflammation index, SJC = swollen joint counts, TJC = tender joint counts, VAS = visual analogue scale.

**Table 3:** Comparison of the differences in clinical indicators and hematological indices between active and remission RA patients.

Variables	Active group (97)	Remission (35)	*P*-value
Age (yr)	60 (52, 71)	52 (44.5, 59)	.003
Disease duration (yr)	3 (1, 6)	5 (2, 5.5)	.342
SJC	2 (0, 5)	0 (0, 0)	<.001
TJC	3 (2, 5)	0 (0, 1)	<.001
CRP	7.91 (2.53, 22.5)	1.3 (0.635, 2.945)	<.001
ESR	32 (21, 53)	10 (7, 13.5)	<.001
VAS	30 (20, 50)	10 (10, 10)	<.001
DAS28-ESR	4.15 (3.27, 5.03)	1.94 (1.645, 2.16)	<.001
DAS28-CRP	3.46 (2.75, 4.46)	1.57 (1.305, 2.015)	<.001
Anti-CCP	200 (30.1, 200)	127 (9.535, 200)	.319
RF	107.7 (50.2, 172.9)	63.9 (13.3, 110.45)	.002
WBC	6.72 (5.36, 8.22)	5.48 (4.235, 7.545)	.015
PLR	144.38 (113.89, 207.27)	149.23 (111.92, 168.78)	.456
SII	632.7 (415.25, 1072.2)	482.14 (337.57, 745.22)	.017
MPV	8.8 (8, 9.225)	9 (8.7, 9.65)	.043
PLT	259 (214, 305)	224 (191, 261.5)	.013

Anti-CCP = anti-cyclic citrullinated peptide antibody, CRP = C-reactive protein, DAS28 = Disease Activity Score 28 joints, ESR = erythrocyte sedimentation rate, MPV = mean platelet volume, PLR = platelet-to-lymphocyte ratio, PLT = platelet, RA = rheumatoid arthritis, RF = rheumatoid factor, SII = systemic immune-inflammation index, SJC = swollen joint counts, TJC = tender joint counts, VAS = visual analogue scale, WBC = white blood cell.

### 3.4. Comparison of hematological indices between remission and active RA patients

According to DAS28-ESR, 132 patients with RA were divided into 2 groups: active group (DAS28-ESR > 2.6) and remission group (DAS28-ESR ≤ 2.6) (Table 3). There were 97 patients with active RA and 35 with RA remission. The mean ages of active RA patients and remission RA patients were 60 (52,71) years and 52 (44.5,59) years, respectively. Statistical significance was observed in age, SJC, TJC, CRP, ESR, VAS, DAS28-ESR, DAS28-CRP, RF, WBC, PLT, SII, and MPV (all *P* < .05) between the 2 groups. However, there was no statistical difference in disease duration, anti-CCP antibody level, and PLR between the 2 groups (Table 3).

### 3.5. ROC curves of SII, PLR, and MPV for the predict of the disease activity of RA patients

**Figure 1.**
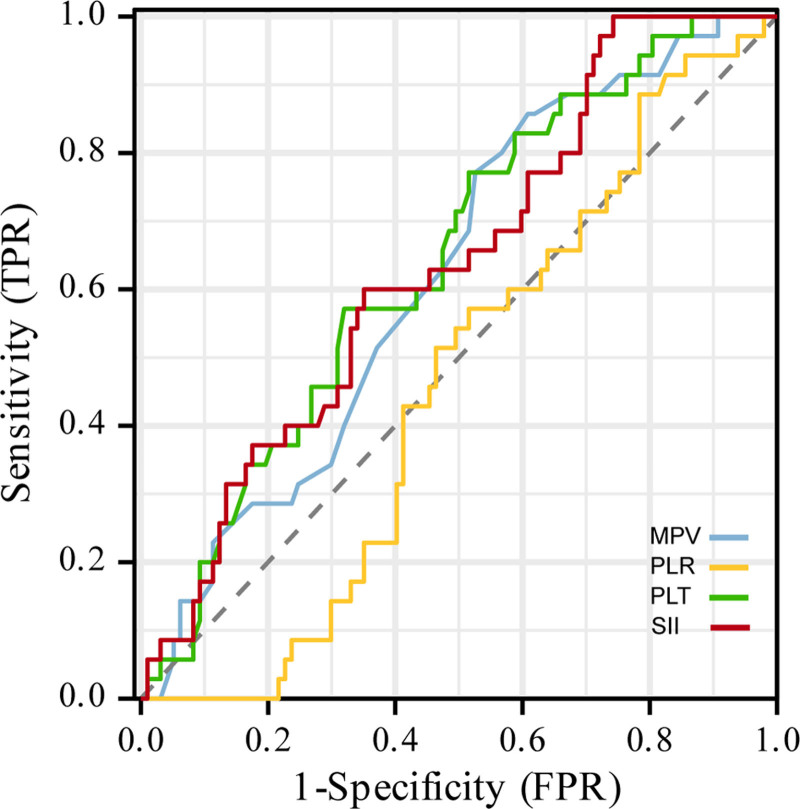
Receiver operating characteristic curves of PLR, SII, and MPV for differentiating the disease activity in patients with RA.21q. MPV = mean platelet volume, PLR = platelet–lymphocyte ratio, RA = rheumatoid arthritis, SII = systemic immune-inflammation index.

ROC curve analysis was performed to assess the ability of these hematological indices to discriminate patients with active RA from those in remission (Table 4, Fig. 1). The area under the curve of SII, PLR, PLT and MPV were 0.636 (95%CI:0.533, 0.739), 0.457 (95%CI:0.356, 0.558), 0.643 (95%CI:0.541, 0.744) and 0.620 (95%CI:0.518, 0.722). The optimal threshold for PLT was 262.5, with a sensitivity of 77.1% and specificity of 48.5%. The optimal cutoff level of the SII was 1057.8, with a sensitivity of 100% and a specificity of 25.8%. The optimal clinical cutoff level of MPV was 8.45, with a sensitivity of 85.7% and specificity of 39.2%.

**Table 4:** ROC analysis of the studied parameters for discriminating active RA from remission RA.

	Cutoff value	Area	Sensitivity	Specificity	95% CI		Youden index
					Lower bound	Upper bound	
PLR	102.01	0.457	0.886	0.217	0.356	0.558	0.102
MPV	8.45	0.620	0.857	0.392	0.518	0.722	0.249
SII	1057.8	0.636	1	0.258	0.533	0.739	0.252
PLT	262.5	0.643	0.771	0.485	0.541	0.744	0.256

MPV = mean platelet volume, PLR = platelet-to-lymphocyte ratio, PLT = platelet, RA = rheumatoid arthritis, ROC = receiver-operating characteristic curves, SII = systemic immune-inflammation index.

## 4. Discussion

To our knowledge, this study represents the first investigation into the potential utility of readily available hematological parameters –PLR, systemic immune-inflammation index (SII), and MPV) – for assessing disease activity in RA. Our findings demonstrate distinct alterations in these parameters in RA patients compared to HCs and suggest that SII and MPV, in particular, correlate with established disease activity measures and may aid in distinguishing active disease from remission

The study showed significant elevation in PLT in RA patients versus HCs. Corroborating earlier findings,^[12]^ PLT counts are significantly increased in active RA compared to remission RA in this study. These alterations likely reflect underlying inflammatory processes involving platelet activation, consumption, and potential alterations in lymphocyte subsets driven by the disease.^[13,14]^ Diverse inflammatory conditions activate circulating platelets, which in turn drive the production and release of pro-inflammatory cytokines (e.g., interleukin-1). The MPV, reflecting platelet size, shows conflicting associations with RA in the literature. Yazici et al^[15]^ reported higher MPV values in RA patients, correlating with inflammatory markers (ESR, CRP) and disease activity (DAS28). Whereas, other studies^[16]^ indicate that active RA is associated with smaller platelet sizes compared to remission, and Şahin et al^[17]^ observed reduced platelet counts in RA patients. Our findings align with this latter perspective. We observed significantly lower MPV levels in RA patients versus HCs, with even lower MPV in active RA compared to remission. Furthermore, MPV demonstrated a negative correlation with DAS28-CRP, ESR, and CRP levels. This decrease in MPV may reflect increased platelet consumption secondary to inflammation.

The systemic immune-inflammation index (SII), derived from platelet, neutrophil, and lymphocyte counts (PLT × N/L), serves as an indicator of systemic inflammation and disease activity in inflammatory rheumatic diseases like AOSD.^[18]^ Evidence supports the utility of this indicator in predicting mortality for cancer patients.^[19]^ Other researchers view it as a potential indicator for tracking the progression of a certain disease. However, SII research in RA is scarce. Our study revealed that RA patients had higher SII than HCs, with active disease patients showing greater levels than those in remission. Elevated SII reflects heightened immune activity that may modulate RA pathogenesis. We also observed associations between SII and both DAS28-ESR and DAS28-CRP, corroborating findings by Choe et al,^[20]^ indicating its potential diagnostic utility in RA. The significant differences found specifically for SII between active RA patients and those in remission further support their connection to the intensity of the inflammatory state.

Notably, while PLR was elevated in RA overall and correlated with disease activity scores (DAS28-ESR and DAS28-CRP), it did not significantly differentiate between active and remission states in this cohort, suggesting it may be less sensitive than SII or MPV for specifically identifying active disease flares, which is compatible with other studies.^[21,22]^

The correlation analysis provides crucial insights. The positive correlations of both SII and PLR with DAS28-ESR and DAS28-CRP underscore their association with the overall burden of inflammation and joint disease activity in RA. The negative correlation observed between MPV and DAS28-CRP is particularly interesting. A lower MPV in active disease may indicate increased platelet turnover and consumption during heightened inflammation, releasing smaller, younger platelets into circulation. The lack of significant correlation between MPV and DAS28-ESR warrants further investigation but could potentially relate to differences in the biological processes reflected by CRP (acute-phase reactant) versus ESR (influenced by multiple factors including anemia and immunoglobulin levels). The ROC curve analysis strengthens the clinical relevance of our findings. The demonstrated value of SII and MPV in differentiating active RA from remission highlights their potential as accessible, cost-effective adjunctive tools for disease activity assessment.

## 5. Conclusions

In summary, our results indicate that the systemic immune-inflammation index (SII) and MPV are significantly associated with disease activity in RA, as measured by DAS28. SII shows consistent elevation and correlation with activity, while a decreased MPV appears linked to active inflammation, particularly associated with CRP levels. Both indices demonstrated potential in distinguishing active disease from remission. These readily available complete blood count-derived biomarkers, especially SII and MPV, show promise as practical and complementary tools to aid clinicians in evaluating and monitoring disease activity in patients with RA.

## 6. Limitations of this study

Our study has some limitations. First, this study was retrospective and conducted at a single center; therefore, the results may be relatively weak. Furthermore, all clinical data and laboratory indicators were collected from electronic medical records, which were measured at different times, resulting in poor consistency. Furthermore, the number of samples was relatively small, and some patients were rolled out due to missing clinical data, which could have led to selection bias. Therefore, further multicenter prospective studies are needed to confirm our results.

## Author contributions

**Conceptualization:** Haihua Wu, Xiufang Chen.

**Data curation:** Haihua Wu, Dan Yin.

**Formal analysis:** Dan Yin.

**Funding acquisition:** Haihua Wu, Xiufang Chen.

**Investigation:** Haihua Wu, Xiufang Chen.

**Methodology:** Haihua Wu, Dan Yin, Chenyang Ma.

**Project administration:** Haihua Wu, Xiufang Chen.

**Resources:** Chenyang Ma.

**Software:** Chenyang Ma.

**Supervision:** Xiufang Chen.

**Writing – original draft:** Haihua Wu, Dan Yin.

**Writing – review & editing:** Xiufang Chen.
